# Community-based prevention leads to an increase in condom use and a reduction in sexually transmitted infections (STIs) among men who have sex with men (MSM) and female sex workers (FSW): the Frontiers Prevention Project (FPP) evaluation results

**DOI:** 10.1186/1471-2458-10-497

**Published:** 2010-08-18

**Authors:** Juan-Pablo Gutierrez, Sam McPherson, Ade Fakoya, Alexander Matheou, Stefano M Bertozzi

**Affiliations:** 1 Division of Health Economics, Centre for Evaluation Research & Surveys, National Institute of Public Health, Mexico; 2 Health Policy Unit, London School of Hygiene & Tropical Medicine; 3 Field Programmes & Planning Analysis and Learning (PAL), International HIV/AIDS Alliance, Brighton, UK; 4 International HIV/AIDS Alliance, Brighton, UK; 5 India HIV/AIDS Alliance, New Delhi, India

## Abstract

**Background:**

India has an estimated 2.0 million to 3.1 million people living with HIV; it has the highest number of HIV-positive people in Asia and ranks third in the world. The Frontiers Prevention Project (FPP) was implemented in 2002 to conduct targeted prevention intervention geared towards female sex workers (FSW) and men who have sex with men (MSM) in the state of Andhra Pradesh (AP). This paper reports the overall changes in behaviour and STI outcomes between 2003/4 and 2007 and also describes the changes attributed to the FPP.

**Methods:**

The evaluation used two cross-sectional surveys among MSM and FSW at 24 sites in AP. Surveys were implemented using a similar methodology. Univariate analyses were conducted by comparing means: baseline vs. four-year follow-up and FPP vs. non-FPP. For both MSM and FSW, random and fixed-effects logit regression models at the site level were estimated for *condom use with last partner*, *syphilis sero-positivity *and *HSV 2 sero-positivity*. In addition, for FSW we estimated models for *condom use with regular partner*, and for MSM we estimated models for *condom use with last female partner*.

**Results:**

Among MSM, fixed-effects analysis revealed that FPP was positively correlated with the probability of *condom use with last female sexual partner *and negatively correlated with the individual probability of *sero-positivity to syphilis and HSV 2*. Among FSW, the FPP intervention was significantly correlated with increased *condom use with regular partners *and with lower probability of *STI sero-positivity*.

**Discussion:**

Important changes in behaviours related to an increase in prevention activities translated to reductions in STI sero-prevalence in AP, India. In contrast with non-FPP sites, the FPP sites experienced an intense community approach as part of the FPP intervention, and the general increase in condom use and its effect on STI sero-prevalence reflected the efficacy of these intense prevention activities focused on key populations in AP.

## Background

India has a reported 2.0 to 3.1 million people living with the human immunodeficiency virus (HIV), representing the highest number of HIV positive people in Asia and third in the world. The overall HIV prevalence in India is 0.36%, a figure below the WHO/UNAIDS threshold of 1% for generalised epidemic. The high reported HIV prevalence among some high-risk groups such as female sex workers (FSW) and men who have sex with men (MSM) (above 5%), puts the country in the classification of concentrated epidemic. Protecting India's large population of FSW and MSM from infection is a critical priority in its own right, and it is also an effective way to protect the remaining population from infection, highlighting the importance of enduring effective prevention for these populations [1,2].

Andhra Pradesh (AP), with a total population of 76.2 million, is located in the south-eastern part of India. This state reported one of the fastest increasing HIV/AIDS rates in India when the FPP began and is among the six states in India with the highest prevalence. About 10% of all AIDS cases in India are in AP. In 2005 the HIV prevalence in ante-natal clinics (ANC) was estimated to be 2%, and the prevalence at sexually transmitted infections (STI) sentinel clinics was 22.8%. The wide distribution of the epidemic is reflected in the finding that 19 out of 23 districts in AP had a prevalence exceeding 1%. The sentinel surveillance centre data suggest that the epidemic is not limited to urban areas and that there is little difference between the prevalence rates of migrants and non-migrants [3].

The Frontiers Prevention Project (FPP) was implemented in 2002 by the International HIV/AIDS Alliance to conduct targeted prevention interventions for key populations (KP), defined as those at high risk for HIV infection and transmission, in the state of Andhra Pradesh. Because of their central role in the Indian epidemic, FSW and MSM, together with injection drug users (IDU) and people with HIV (PWH), were defined as KP for the project. The FPP set out to empower KP by improving advocacy within these groups, changing policies that affect these groups, and increasing community awareness. These efforts, combined with the provision of a comprehensive package of prevention interventions implemented on the appropriate scale, aimed to reduce risk-taking behaviours and STI incidence, thereby resulting in a lower HIV incidence among KP, and secondarily among the general population. Additionally, the targeted populations were involved in program planning, and dissemination was intended to increase community ownership of the program and thus its sustainability after the FPP finished. The *prospective *external evaluation described here only evaluated impact among MSM and FSW because of the impossibility of identifying IDU and PWH at baseline, in advance of project implementation [4].

**Figure 1:**
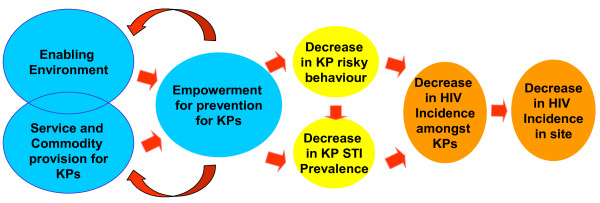
**The FPP theory of change**.

The FPP was based on a theory of change (see Figure 1): In countries with concentrated epidemics, well-defined sets of interventions, focused on key populations affected by the epidemic, would result in reduced incidence of HIV amongst these populations and would also result in curbing of the potential spread of the epidemic into the broader population. The goal was to ensure an environment in which prevention was feasible and adequate services (STI clinics, drop-in centres) and commodities (condoms, lubricants, STI treatment) for prevention were available for key populations. Empowerment of the KP was believed to improve access by prevention workers to the KP, as well as access to and utilization of prevention services by the KP. In turn, risky behaviour and STI prevalence would decrease, and subsequently, there would be a reduction in HIV incidence among KPs and in the broader community [5-7].

As part of the project, an impact evaluation was designed to measure the impact of FPP prevention interventions among FSW and MSM in Andhra Pradesh. Although the original evaluation underwent major changes while the program was being implemented, the final prospective impact evaluation reported dramatic results and may provide useful lessons for other large-scale programs seeking to incorporate prospective impact evaluations into their design.

The original evaluation design was a classic cluster-randomised trial design with 24 geographically distinct sites randomised as either FPP sites that would receive the full FPP prevention program or as non-FPP sites that would receive no intensive community-based prevention interventions beyond the government's existing HIV prevention program. Closely matched sites were chosen for evaluation within a universe of sites that were both operationally feasible and where HIV risk reduction was a priority. Within-pair randomisation was conducted to determine which site would receive the intervention and which would serve as comparison. Extensive community mapping of MSM and FSW at baseline was included in the evaluation, which was used, *inter alia*, to estimate the size of the two populations [8].

The ultimate goal of the program was to reduce HIV incidence; however, when the FPP began it was not possible to include HIV incidence as an endpoint in the evaluation because of the perception by both the government and the implementing organization that it was unethical to do so and that it would adversely affect the prevention program's acceptability in the community. As discussed below, those views evolved over the course of the project. As a result, the original evaluation design included repeat, cross-sectional, anonymous surveys of MSM and FSW that collected socio-demographic information, limited sexual history, detailed risk behaviour information about their last three sexual relations, and samples of blood and urine to test for herpes simples virus type 2 (HSV 2), syphilis, gonorrhoea and Chlamydia.

While the baseline survey was being conducted, the Bill and Melinda Gates Foundation (Foundation) and the AP State AIDS Control Society (APSACS) decided to dramatically increase the scale of prevention activities in the state as part of the Avahan prevention program. The Avahan program, with the goal of saturating key populations with prevention interventions, was very similar in design to the FPP. The most important design difference between the programs was the degree to which each individual community participated in determining which prevention services were of highest priority for that community; community participation was a prominent feature for the FPP but not for Avahan. Because Avahan and APSACS aimed to cover 100% of AP, the original FPP comparison communities became Avahan intervention communities very shortly after the FPP initiated its programs at its intervention sites. Further complicating the situation, the International HIV/AIDS Alliance (Alliance) was the implementation partner for Avahan at many sites in AP [9].

Faced with the loss of the original evaluation design, the decision was made to proceed with a modified design that took advantage of (i) the exogenous variability resulting from the difference between the FPP and Avahan programs in the degree of community participation and (ii) the endogenous variability in program efforts, spending and coverage across program sites, reflecting both site-specific differences as well as differences among the NGOs selected to implement the prevention interventions at the different sites.

The aims of this paper are to describe the socio-demographic characteristics of the FSW and MSM in AP as well as to report the results of the estimated effectiveness of the FPP by using a dosage analysis that takes advantage of the baseline and follow-up survey implemented at 24 sites.

## Methods

The analysis was a site-level panel analysis that used repeated cross-sectional surveys of FSW and MSM from the same geographic sites and followed similar protocols for identifying hotspots and recruiting participants.

Analyses of the baseline surveys have been widely published and are not presented here [10,11]. Results presented here were limited to analyses comparing the MSM and FSW populations at baseline with the MSM and FSW populations at the same 24 sites four years later. Because individual identifiers were not collected, we designed the analysis as a comparison of two cross-sectional surveys conducted at the same sites with similar methodology (i.e., mapping hotspots in each site and key populations surveyed from those spots). Only the baseline data from the sites surveyed in the follow-up were included, and only the HSV 2 and syphilis biological endpoints were included.

### Sampling

Among the original 24 baseline sites, 62 sub-sites (limited by the administrative definition of geographic areas within a district) were described by the type of program implemented (FPP, Avahan (either implemented by Alliance partners or other CBOs, or neither Avahan nor FPP) and by the estimated size of the key populations as reported by the implementing NGOs. Using this dataset and the constraint that the follow-up surveys could be implemented at only 24 sub-sites, a subset of 12 FPP and 12 non-FPP/non-Avahan (referred here after as non FPP) sub-sites was randomly selected; the selection probability was proportional to the size of the MSM and FSW populations at the sites. At these 24 sub-sites, a follow-up survey was implemented and capillary blood was collected for HSV 2 and syphilis testing.

Sample size was calculated to detect a difference in STI prevalence between FPP and non-FPP sites (assuming 12.5% prevalence at non-FPP sites and 9% prevalence at FPP sites) with an intra-cluster correlation of 0.05, resulting in 200 individuals per KP per site based on previous studies [12-14].

Working closely with the community-based organisations (CBOs) in charge of the implementation, we identified the survey hotspots for MSM and FSW. The methodology for this mapping followed the participatory learning and action tools developed by the IHAA [8,15]. The size of each hotspot was estimated based on the individuals present at the time of the visit. To survey FSW, within each hotspot a subsample was defined based on a defined sample by sub-site; because of the smaller number of MSM frequenting the hotspots, all MSM present at the hotspot were asked to participate in the survey.

While the mapping intended to be as comprehensive as possible of each target population, the major limitation when surveying hidden populations is that the sampling universe is restricted to those frequenting sampling points (hotspots in our case) identified in the mapping. As the mapping was implemented systematically and observed by the researchers, there was not an evident bias in this procedure.

While there is no reason to believe that some types of hotspots were systematically excluded, there is an expected sampling bias against MSM and FSW who do not frequent MSM or SW venues. This expected bias is not of concern with respect to estimating the impact of the FPP on the targeted populations because the interventions were also targeted at the KP who frequent sites, but it does not reflect the impact on the excluded populations.

To test for potential biases in the population size estimates generated by the CBO's, an additional mapping exercise was carried out just before each survey by the agency in charge of the survey, with estimates comparable to the ones provided by the participatory mapping.

### Descriptive analysis

Univariate analyses were conducted by comparing simple means: baseline vs. four-year follow-up and FPP/Alliance vs. non-FPP sites. This analysis was conducted to provide a description of the populations at the baseline and follow-up surveys in terms of their socio-demographic characteristics as well as the variables defined below for the impact analysis.

Demographic characteristics reported included age, whether participants were from the same *mandal *(the administrative division below district in AP) as the one in which they were residing at the time of the interview (as a measure of migration), literacy rates, and living arrangements.

For the follow-up, variables related to the intervention were also examined. The degree of satisfaction with the prevention activities (support groups and condom demonstrations) was estimated by asking participants to rate their satisfaction on a 1-to-10 scale (where 10 was defined as the best). In addition, we assessed the knowledge of Mythri condoms, a product developed by Alliance India for its activities.

### Impact analysis

Multivariate regression analyses took into account differences in intervention coverage as measured by whether the respondent had been exposed to Mythri condom distribution, had participated in a peer-education activity, and/or had visited a community prevention centre. For these estimations, an intervention variable was defined by the site category (whether the site was FPP or non-FPP), and the dosage indicators (coverage) were included to control for the heterogeneity in implementation at the FPP sites and the lack of systematically collected data on prevention activities at the non-FPP sites.

For both MSM and FSW, random and fixed-effects logit regression models at the site level were estimated for *condom use with last partner*, *syphilis sero-positivity *and *HSV 2 sero-positivity*. For FSW we also estimated models for *condom use with regular partner*, and for MSM we estimated models for *condom use with last female partner*.

Random-effects models require additional assumptions compared to fixed-effects models, but the estimation of time variant characteristics is more efficient. If the assumption that errors are not correlated with the independent variables (that is needed for the random-effects models) is not met, then the estimator would be inconsistent and the fixed-effects models would be preferred. As the panel was constructed at the site level, is at this level where the assumption needs to hold. The Hausman test was implemented, but because its results were not conclusive, both random effects and fixed effects models were reported [16].

### Outcome variables

As mentioned above, the outcome variables were both behavioural and biomarker-based. Reported condom use was used as the key behavioural indicator, divided for FSW between clients and regular partners and for MSM between male and female partners. In all cases, the variable was defined as 1 when individuals reported using a condom with each of the defined type of partner and 0 when individuals reported not using a condom for anal or vaginal penetrative sex during the last intercourse.

Biomarkers used were sero-positivity for syphilis and sero-positivity for HSV 2. ELISAs were conducted with the eluted blood from the dried capillary blood spots collected during the survey using the positivity cut-off points recommended by the test manufacturers. The HSV 2 test was the HerpeSelect 2 from Focus Diagnostics, and the syphilis test was the Trepanostik™from bioMerieux.

While syphilis is a treatable infection and changes in sero-prevalence are expected to occur due to increased access to treatment, we included HSV 2 as a variable because of its use as a marker for HIV risk, as documented in other studies [17-20]. Assuming that the population would remain unchanged, there was no expectation of change in this indicator, but a reduction in new infections combined with any population change would result in a decrease in the overall prevalence of HSV 2.

### Intervention variables

In all models, the FPP effect was estimated as the interaction between time (baseline or follow-up) and intervention (FPP or non-FPP). Using this interaction, a time trend was estimated in addition to a variable that indicated differences between FPP and non-FPP sites as for the baseline. Note that the FPP vs. Non-FPP comparison is intended to capture the difference in prevention approaches that do or do not include extensive community participation in the design of the package of interventions being implemented in the community. The coverage variables capture the difference in dose of prevention interventions provided in the community.

### Control variables

To control for the differential exposure to the interventions as well as for interventions that occurred in the non-FPP sites, a set of coverage variables was generated from the collected information. Coverage was defined as the percentage of the individuals from a given site who reported participation in key activities for prevention; i.e., they participated in a condom use demonstration, were reached by a peer-educator, and visited a drop-off centre for STI treatment. Because this information was not available from the baseline questionnaire, for analytical purposes, it was assumed that coverage was identical in all sites and close to zero. This assumption was made because prevention activities were described by the baseline site assessment process as not very extensive. However this assumption affects the interpretation of the coefficients of these variables, which were therefore only useful for determining whether the FPP approach had a significant & positive incremental impact; their absolute values are not interpretable.

For MSM, age at the time of the survey, experience in commercial sex (1 if yes, 0 if no), whether family was aware of their sexual behaviour (1 if yes, 0 if no), and ever having sex with a female partner (1 if yes, 0 if no) were included as control variables. For FSW, additional control variables were age at the time of the survey, age at the first commercial intercourse, and whether family was aware of their commercial sex work (1 if yes, 0 if no).

### Ethical approval

The FPP evaluation was approved by the Ethics Committees of Mexico's National Institute of Public Health, the Administrative Staff College of India, and the International HIV/AIDS Alliance. Clearance for the study was provided by the Indian Health Ministry's Screening Committee, Indian Council of Medical Research, New Delhi.

## Results

The intervention was implemented by 14 NGOs at 26 sites in 9 districts across the Rayalseema and Telengana regions of AP. By April 2007, 8757 FSW, 5597 MSM and transgender individuals, 4730 PWH and 350 IDU were registered and were receiving health-related services regularly. These services included STI services, behaviour change communication, condom programs, community mobilisation, and enabling and structural interventions. In addition, there was an emphasis on social capital building, network and support formation, empowerment, violence reduction, referrals for HIV testing and basic AIDS care services.

Project-operated STI clinics were set up at all sites, and the medical officers were trained in syndromic case management and basic AIDS care. By April 2007, 6328 FSW, 3136 MSM and 2363 PWH had been given STI treatment; in addition, 3161 FSW, 2086 MSM and 481 PWH received treatment for asymptomatic gonococcus and/or Chlamydia infections. Outreach was performed through a team of outreach workers and peer educators (selected from the community). Condoms were distributed to the KP through the outreach team as well as from the clinic. In total, 3.8 million free condoms were distributed, and 600,000 were distributed through social marketing. Support groups were formed, especially in the Karimangar and Khammam districts, amongst PWH. A state-level sex-worker network was formed.

Overall, 2,786 MSM were interviewed at the 24 sub-sites at baseline: 1,680 at the FPP sub-sites and 1,106 at the non-FPP sub-sites. During the follow-up survey, the team was able to identify a much smaller number of MSM at the non-FPP sub-sites. Only 218 MSM were interviewed at the non-FPP sub-sites and 1,317 MSM at the FPP sites (total sample size in the follow-up was 1,535). At the same sites at baseline, 3,442 FSW were interviewed: 1,692 in FPP and 1,750 in non-FPP sites. During the follow-up survey, 1,292 FSW were interviewed at the FPP sites and 855 at the non-FPP sites.

Table 1 and Table 2 describe sample characteristics in both surveys (baseline and follow-up) of MSM and FSW, respectively. Basic demographic characteristics were similar between individuals in both surveys. For the analysis, we included only individuals in the follow-up survey who were likely to have been at the sites at baseline, i.e., people who reported living or working in the area for at least four years.

**Table 1:** Characteristics of MSM participants in the survey by type of intervention and time (average and 95% CI)

Characteristics	Pre-intervention survey		Post-intervention survey	
	
	NFPP Mean	FPP Mean	NFPP Mean	FPP Mean
Age	27.71 (20.10-29.32)	28.57 (27.95 -29.19)	31.45 (+) (29.14-33.76)	29.14 (27.76-30.52)

Participants from the same village where they were interviewed	90% (85% -95%)	96% (94%-0.98%)	92% (86% -98%)	95% (93% -98%)

Participants from the same mandal in which they were interviewed	82% (68% -96%)	89% (77%-100%)	96% (85%-100%)	90% (77%-100%)

Participants who were currently in a relationship with another man	90% (98%-100%)	100% (99-100%)	100%	99% (99-100%)

Participants currently living with a male partner	5% (1% -9%)	6% (3%-10%)	7% (2%-15%)	17% **(+)** (8%- 26%)

Participants married to a male	5% (3% -7%)	5% (2% -8%)	10% (2% -17%)	16%**(+)** (9%-22%)

Participants whose families were aware of their sexual preferences	35% (25% -44%)	48% (*) (40% -56%)	75%**(+)** (58% -93%)	17%**(+)** (8%-26%)

Participants who used lubricants	46% (42% -51%)	53% (40% -66%)	30% **(+)** (26% -33%)	33%**(+)** (22%-44%)

Participants who reported participation in commercial sex	19% (10%-28%)	22% (17%-27%)	51%**(+)** (33% -68%)	42%**(+)** (36%-48%)

Participants who reported that at least one of the 3 last sexual intercourses was commercial	13% (7% -19%)	19% (14% -25%)	44%**(+)** (27% -60%)	39%**(+)** (35%-44%)

Participants who used a condom during their last sexual encounter	77% (54%-100%)	79% (50%-100%)	90% (80% -99%)	94% (92% -96%)

Participants who have had a sexual relation with a female	73% (66% -80%)	72% (66% -79%)	86%**(+)** (76% -97%)	83% (72% -93%)

Participants who used a condom with their last female partner	14% (9% -18%)	15% (7% -23%)	32%**(+)** (30% -34%)	40%**(+)** (32% -49%)

Participants who were sero-positive for syphilis	20% (10% -30%)	22% (14% -30%)	9% (3% -16%)	12%**(+)** (5% -19%)

Participants who were sero-positive for HSV 2	34% (19% -49%)	40% (32% -47%)	29% (16% -43%)	32% (27% -37%)

Participants who received treatment for their last STI episode	80% (75% -84%)	98% (*) (95%-100%)	100%**(+)**	100%**(+)**

Participants who had an HIV test	12% (7% -17%)	9% (4% -14%)	52% **(+)** (42% -62%)	54% **(+)** (50% -59%)

Number of male partners in the last 4 weeks	3.72 (3.11-4.33)	5.75 (*) (3.97-3.53)	6.83 **(+)** 5.62-8.05)	7.51 (6.08- 8.95)

Participants who had attended a condom demonstration			62% (53% -71%)	66% (59% -73%)

Average rate of condom demonstration attendance			**7.86** **(7.69-8.03)**	8.50 (*) **(8.04-8.96)**

Participants who had been contacted by a peer educator			53% (36%-70%)	52% (36%-68%)

Participants who had visited a drop-in centre			42% (13%-72%)	54% (40%-68%)

* p < 0.05 FPP vs NFPP; + p < 0.05 baseline vs. follow-up

**Table 2:** Characteristics of FSW participants in the survey by type of intervention and time (average and 95%CI)

Characteristics	Pre-intervention survey		Post-intervention survey	
	
	NFPP	FPP	NFPP	FPP

Age	27.82 (27.29- 28.35)	27.61 (26.92 -28.30)	29.67(+) (28.87-30.48)	29.92(+) (29.09-30.76)

Participants from the same village where interviewed	90% (83% -97%)	96% (92% -99%)	100%(+) (99%-100%)	100%(+) (99%-100%)

Participants from the same mandal where interviewed	83% (73% -94%)	60% (34% -85%)	98% (+) (97% -98%)	100% (+.*)

Participants who are married	10% (7% -13%)	18% (8% -29%)	4% (+) (1% -6%)	13% (2%-27%)

Participants who have participated in a support group	13% (3% -23%)	10% (1% -19%)	66%(+) (45% -88%)	76%(+) (65%-87%)

Participants whose families are aware of their sexual work	33% (24%-42%)	30% (19%-41%)	45% (32%-58%)	49% (+) (33%-65%)

Participants who used a condom with their last client	68% (55% -80%)	72% (62% -82%)	98% (+) (97%-100%)	99% (+) (98%- 100%)

Participants who have a regular partner	56% (50% -63%)	59% (48% -70%)	90%(+) (82% -97%)	83%+ (74% -92%)

Participants who used a condom with their regular partner	5% (2% -8%)	7% (4% -10%)	21%(+) (14% -28%)	38% (+) (16% -60%)

Participants who received treatment for their last STI episode	90% (86% -95%)	84% (78% -90%)	94% (91% -97%)	95% (+) (92% -98%)

Participants who have had an HIV test	7% (4% -11%)	13% (8% -19%)	87%(+) (82% -92%)	90%(+) (85% -96%)

Participants who are sero-positive to syphilis	13% (7% -19%)	18% (11% -25%)	8%(+) (4% -11%)	10%(+) (9% -12%)

Participants who are sero-positive to HSV 2	46% (25% -66%)	47% (32% -62%)	21% (13% -29%)	29%(+) (14% -44%)

Average age of first commercial intercourse	23.76 (23.32 -23.32)	23.13 (22.13-22.13)	23.92 (23.12-23.12)	23.32 (21.82-21.82)

Average age of first sexual intercourse	15 (14.87-15.13)	15.30 (14.89-15.71)	16.54(+) (16.28-16.80)	16.41 (15.38-17.44)

Participants who have participated in a condom demonstration			74% (59%-90%)	88% (75%- 100%)

Participants who know about Mytrthi condoms			10% (10% -18%)	9% (3% -15%)

Average satisfaction with support groups			7.56 (6.77-8.35)	8.27 (7.75-8.79)

Average rate of condom demonstrations			7.37 (6.63- 8.12)	8.45(*) (7.85- 9.05)

Average number of clients in the last week			9.90 (6.72-13.09)	9.12 (6.32-11.92)

Participants who have visited a drop-in centre			30% (11% -49%)	68%(*) (54% -81%)

* p < 0.05 FPP vs NFPP; + p < 0.05 baseline vs follow-up

Table 1 also compares the outcome variables between baseline and follow-up for MSM. Note that the percentage of MSM who reported *condom use with last partner *increased for both non-FPP and FPP sites between baseline and follow-up (from 77% and 79% to 90% and 94%, respectively), and the percentage of MSM who reported condom use with last female partner also increased but remained low at non-FPP and FPP sites between baseline and follow-up (from 14% and 15% to 32% and 40%, respectively). There was an impressive decrease of syphilis sero-prevalence between baseline and follow-up in both non-FPP and FPP sites (from 20% and 22% to 9% and 12%, respectively); similar trends were seen for HSV 2 sero-prevalence (from 34% and 40% to 29% and 32%, respectively).

We compared the outcome variables between baseline and follow-up for FSW in Table 2. *Condom use with last client *increased for non-FPP and FPP sites between baseline and follow-up from 68% and 72% to 98% and 99%, respectively. While *condom use with regular partners *also increased for non-FPP and FPP sites between baseline and follow-up (from 5% and 7% to 21% and 38%, respectively), unprotected sex with these partners remained high. As among MSM, biomarkers from FSW also showed an impressive reduction, with syphilis sero-prevalence dropping in both non-FPP and FPP sites from 13% and 18% at baseline to 8% and 10% at follow-up; HSV 2 sero-prevalence decreased at the FPP sites from 46% at baseline to 33% at follow-up but increased in the non-FPP sites from 47% at baseline to 49% at follow-up. With regard to prevention activities, an important increase in FSW participation in support groups occurred for both FPP and non-FPP sites (from 10% at baseline to 76% at follow-up and from 13% at baseline to 66% at follow-up, respectively).

**Figure 2:**
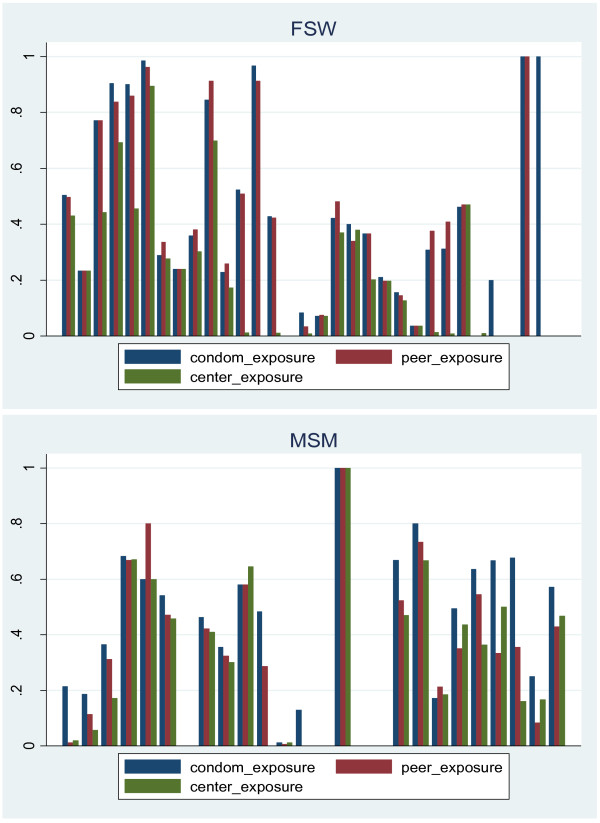
**Dosage measures distributed across sites (histogram)**. Bottom: The x-axis represents sites and the y-axis represents percentages.

**Figure 3:**
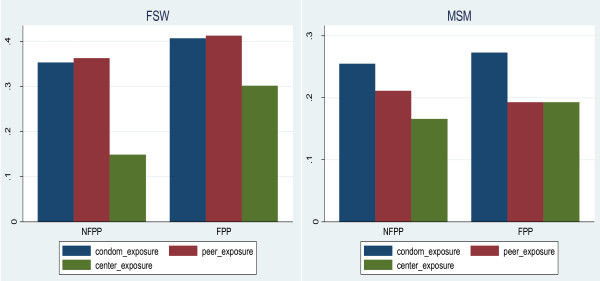
**Dosage measures by intervention (FPP vs. non-FPP sites) (histogram)**. Bottom: The y-axis represents the average site percentages for each dosage measure.

Dosage was highly heterogeneous across sites, ranging from none to 90% for the average measure (simple mean of the 3 indicators). Figure 2 shows the distribution of the 3 dosage measures as estimated from the surveys of both MSM and FSW at the site level. Figure 3 shows that because of other prevention efforts implemented in the non-FPP sites, the average coverage of the 3 dosage indicators was similar between FPP and non-FPP sites. While dosage tended to be higher among FPP sites, some FPP sites had low intervention levels (at least according to the dosage indicators used) and some non-FPP sites had higher levels of prevention activities. Overall, the highest exposures were for condom distribution activities, followed by peer contact. Utilization of drop-in centres was lower and possibly related to STI symptoms. These data were used to estimate the effect of prevention activities on the outcome indicators, assuming a direct relationship between intervention intensity and results.

Regression analyses were conducted, adjusting all models for sampling design (using random and fixed-effects models by site). The logistic model results are reported for MSM in Table 3 and for FSW in Table 4. In both cases, all models were also adjusted for age and dosage measures. The outcome variable was the interaction between time and intervention (FPP).

**Table 3:** Effect of FPP on selected outcome variables for MSM in AP, India*

	Used condom with last male sexual partner		Used condom with last female sexual partner		Sero-positivity for HSV 2		Sero-positivity for syphilis	
	**Random effects**	**Fixed effects**	**Random effects**	**Fixed effects**	**Random effects**	**Fixed effects**	**Random effects**	**Fixed effects**

**FPP = 1**	-0.138		0.037		0.658		0.484	

**Follow-up = 1**	1.029	0.908	2.087**	2.268**	-2.154**	-2.012*	-4.092***	-3.798**

FPP * Follow-up	0.246	0.302	0.605	1.196**	-1.368**	-1.482***	-0.748**	-0.687**

**Site average exposure to condom demonstrations**	2.778	2.758	-2.321*	-3.481**	4.024**	4.083**	4.912**	4.445**

**Site average exposure to peer educators**	0.006	0.040	3.052*	4.823**	-1.425	-0.365	-0.684	0.423

**Site average access to drop-in centres**	-0.636	-0.494	-1.049	-1.966	0.094	-1.125	0.756	-0.461

**Age in years**	-0.047	-0.047***	-0.134***	-0.134***	0.029***	0.030***	0.059***	0.058

**At least 1 of 3 last sexual encounters was commercial = 1**	0.114	0.121	-0.4121**	-0.370**	4.63e-06	-0.011	-0.113	-0.118

**Had a female sexual partner = 1**	0.360	0.379**			-0.166	-0.182	-0.4371***	-0.444***

**Constant**	1.272		1.799***		-2.722***		-2.261***	

**N**	2 361	2 361	1 510	1 510	2 624	2 624	2 579	2 579

*** p < 0.001, ** p < 0.05, * p < 0.1. All values from the regressions are the coefficients.

**Table 4:** Effect of FPP on selected outcome variables for FSW in AP, India

	Used condom with last sexual client		Used condom with last sexual regular partner		Sero-positivity for HSV 2		Sero-positivity for syphilis	
	**Random effects**	**Fixed effects**	**Random effects**	**Fixed effects**	**Random effects**	**Fixed effects**	**Random effects**	**Fixed effects**

**FPP = 1**	0.135		-0.187		0.737**		0.673	

**Follow-up = 1**	2.519**	2.212**	1.774**	2.079*	-1.725**	-2.248***	-2.324***	-2.450***

FPP * Follow-up	0.569	0.554	1.091**	1.104**	-0.490*	-0.623**	-0.894***	-0.948***

**Site average exposure to condom demonstrations**	-1.304	4.826	3.011	7.551**	-1.708	-1.150	-7.350***	-7.560***

**Site average exposure to peer educators**	-1.304	-2.287	-3.247	-8.088**	1.955	1.891	6.548***	6.871***

**Site average access to drop-in centres**	-2.002**	-1.863**	-0.231	-0.112	1.745**	2.077***	3.814***	3.932***

**Age in years**	-0.029*	-0.032*	0.024	0.023	0.038**	0.035**	0.025**	0.024**

**Age of first commercial sex**	-0.028	-0.022	-0.021	-0.023	-0.049**	-0.044**	-0.008	-0.007

**Constant**	2.348***		-3.097***		-2.019***		-0.880**	

**N**	3 329	3 084	2 091	2 091	3 603	3 579	3 516	3 516

*** p < 0.001, ** p < 0.05, * p < 0.1. All values from the regressions are the coefficients

The analysis for MSM included as covariates whether the participant reported participating in commercial sex during the three most recent intercourses and whether he had had a female sexual partner. As presented in Table 4, there were significant and positive effects of the FPP compared to the non-FPP interventions for condom use with female partners and for syphilis and HSV 2 sero-positivity. The impact variable (interaction between time and intervention) in the fixed-effects model was positively correlated with the probability of *condom use with last female sexual partner *and negatively correlated with the individual probability of syphilis sero-positivity and HSV 2 sero-positivity. Age was negatively associated with *condom use with regular partners *and positively associated with both syphilis and HSV 2 sero-positivity. We found a significant and positive difference between baseline and follow-up for condom use with a female partner and a negative difference between baseline and follow-up for syphilis and HSV 2 sero-positivity. For the dosage measures, access to condoms paradoxically seemed to have a reverse effect: it was negatively correlated with condom use and positively correlated with STIs. Participation in commercial sex was correlated with lower probability of condom use with female partners. Having had sex with female partners was correlated with a higher probability of condom use with last male partner and lower probability of syphilis sero-positivity.

Table 4 reports the results for FSW. The FPP intervention had a significant effect on condom use with regular partners and on STI sero-positivity. The effect variable was positively correlated with condom use with regular partners in both random and fixed-effects models and negatively correlated with the probability of syphilis and HSV 2 sero-positivity. The trend variable (time) suggested a general increase in condom use with both clients and regular partners as well as a drop in STI sero-prevalence. Age was negatively correlated with *condom use with last client *and with a decline in use for older FSW but not with *condom use with regular partners*. Age was also positively correlated with STI sero-status. Access to condom demonstrations was both positively correlated with condom use and negatively correlated with STIs. Access to peer-education and to drop-off centres was correlated with a higher probability of STIs.

## Discussion

The results presented here highlight some important overall changes in sexual behaviours among MSM and FSW in AP, India, comparing 2 cross-sectional surveys implemented in the same sub-sites with 3 years difference; these changes were associated with changes in STI sero-prevalence: FPP sites over time had increased reductions of STIs among MSM & FSW as compared to non-FPP. The reported trends suggest that important prevention efforts established and implemented in AP effectively modified the risk of STIs, including HIV, among the population vulnerable to the HIV epidemic; the results also suggest that if the proposed theory of change is correct, the prevention efforts also reduced the risk of HIV infection among the general population. Moreover, these results are consistent with those reported for Karnataka (a state bordering AP), where increased safe sexual behaviours were found to be associated with a reduction in STIs [21].

In addition to the overall changes, important differences were noted between the FPP and non-FPP sites. These differences may be attributed to the intensive community approach of the FPP intervention.

These positive results, even considering the limitations of the evaluation design, suggest that a strong community component may significantly potentiate prevention impact. It is all the more convincing because over time Avahan became increasingly focused on community mobilization. While this supports the arguments in favour of community participation, it also calls for more robust evaluation in the future to characterize and quantify the benefits and costs of different approaches for community engagement and mobilization to accompany the provision of prevention services.

There were, however, areas that still require additional attention, areas in which the impact on condom usage was less evident. First, with regard to the female partners of MSM, the reasons for low condom usage varied but one of the primary reasons cited in the qualitative analyses was that many MSM did not disclose their sexual orientation to their wives and therefore struggled to explain the necessity of condom usage. This issue may be particularly difficult to address in places like AP where sterilisation is such a common form of contraception. Therefore, prevention work is handicapped unless it addresses broader issues surrounding sexual identity, stigma and discrimination [2,22]. As reported in other studies, these data reflect the need for prevention activities that address the cultural needs specific to MSM in India [23,24].

Similarly, low condom use by FSW with regular or non-paying partners is a serious concern. Their relationships with these partners are likely to have emotional (trust) and/or economic (security, protection, coercion) characteristics that interfere with regular condom use.

While these areas highlight room for improvement, some increases in condom usage with wives and regular partners and the almost universal condom use with clients by FSW and with male partners by MSM suggest that prevention activities have had a strongly positive effect over the period of observation.

There are, however, some caveats. First, the desired behaviour change was clear to all participants, and more so after three years of prevention promotion in their communities; therefore, the self-reports of condom usage should be treated with caution. Second, the complications of the evaluation design, most specifically the introduction and expansion of the Avahan project, made it difficult to estimate the effects of the FPP because we were not able to differentiate the changes observed in the study from secular trends [9]. In addition, there were important differences in the size of the sample by type of site in the pre and post intervention surveys for MSM. During the post-intervention survey, a much smaller fraction of the expected MSM in the non FPP sites was interviewed. We don't have sufficient data to explain the cause of this significant difference in response rates. It could have to do with the degree to which the FPP NGOs facilitated access to the hotspots in the follow-up survey; it could have to do with some concerns that the MSM population had about participating at the time of the survey, concerns that were less acute in the FPP sites. It is speculative to suggest what direction the smaller sample might bias the outcome measures. If the smaller sample has a higher proportion of the most visible, most at-risk MSM then it could exaggerate the estimated impact. If the same factors that lead the population to be less accessible to the survey are associated with under-reporting of risk, or with reduction of high-risk venues, then the impact might be underestimated.

## Conclusions

The FPP-specific results here strongly suggest that there are important benefits associated with approaches that engage communities in the design and implementation of prevention interventions for their communities. The overall results (the time trends) documents important changes in sexual behaviours and reductions in STI prevalences that accompany the scale-up of community-wide prevention programs that achieve high levels of coverage. These changes are also accompanied by important reductions in HIV prevalence during the same period that has been documented elsewhere.

These results should motivate others to both implement similar comprehensive prevention programs as well as include more robust prospective evaluations in the programs so that the problems of attribution illustrated here can be minimized. It is also important to better evaluate the potential additional effect of the participatory approach, as part of the scale up of prevention programs in the future.

## Competing interests

The authors declare that they have no competing interests, but in the interest of full disclosure, SM, AF and AM are employed by the organization that implemented the FPP program and SMB has recently joined the Bill and Melinda Gates Foundation that funded the FPP and the external evaluation.

## Authors' contributions

JPG participated in the design of the study, performed the statistical analysis, and prepared the manuscript draft. SM prepared the draft for the background section and participated in the design of the study. AF & AM contributed to the analysis. SMB lead the design of the study & participated in the statistical analysis. All authors read and approved the final manuscript.

## Pre-publication history

The pre-publication history for this paper can be accessed here:

http://www.biomedcentral.com/1471-2458/10/497/prepub

## References

[B1] UNAIDS 2.5 million people in India living with HIV, according to new estimates 2007 http://data.unaids.org/pub/PressRelease/2007/070706_indiapressrelease_en.pdf cited 2009 September 18, 2009

[B2] Thomas B Mimiaga MJ Menon S Chandrasekaran V Murugesan P Swaminathan S Unseen and unheard: predictors of sexual risk behavior and HIV infection among men who have sex with men in Chennai, India AIDS Educ Prev 2009 21 4 372 83 10.1521/aeap.2009.21.4.372 19670971 PMC3623672

[B3] APSACS Facts, figures and response to HIV/AIDS in Andhra Pradesh.Hyderabad: Andhra Pradesh State AIDS Control Society 2005

[B4] International-HIV/AIDS-Alliance, ASCI, NIMS, INSP Key indicators for Frontiers Prevention Project: Report on baseline study in Andhra Pradesh, India 2006 Brighton, UK: International-HIV/AIDS-Alliance

[B5] Blankenship KA West BS Kershaw TS Biradavolu MR Power, community mobilization, and condom use practices among female sex workers in Andhra Pradesh, India Aids [Article] 2008 22 S109 S16 10.1097/01.aids.0000343769.92949.dd 19098471

[B6] Blankenship KM Burroway R Reed E Factors associated with awareness and utilisation of a community mobilisation intervention for female sex workers in Andhra Pradesh, India Sexually Transmitted Infections [Article] 2010 86 I69 I75 10.1136/sti.2009.038653 PMC3252621 20167735

[B7] IHAA Summary Project Description-Frontiers Prevention Project: a global initiative to slow the spread of HIV and build capacity for effective and sustainable community responses Brighton, UK 2002

[B8] IHAA Tools Together Now! 100 participatory tools to mobilise communities for HIV/AIDS 2006 Brighton: The International HIV/AIDS Alliance

[B9] Verma R Shekhar A Khobragade S Adhikary R George B Ramesh BM Scale-up and coverage of Avahan: a large-scale HIV-prevention programme among female sex workers and men who have sex with men in four Indian states Sexually Transmitted Infections 2010 2010 86 Suppl 1 i76 i82 10.1136/sti.2009.039115 PMC3252619 20167737

[B10] Dandona L Dandona R Gutierrez JP Kumar GA McPherson S Bertozzi SM Sex behaviour of men who have sex with men and risk of HIV in Andhra Pradesh, India Aids 2005 19 6 611 9 10.1097/01.aids.0000163938.01188.e4 15802980

[B11] Dandona L Dandona R Kumar GA Gutierrez JP McPherson S Bertozzi SM How much attention is needed towards men who sell sex to men for HIV prevention in India? BMC Public Health 2006 6 31 10.1186/1471-2458-6-31 16478546 PMC1421390

[B12] Divekar AA Gogate AS Shivkar LK Gogate S Badhwar VR Disease prevalence in women attending the STD clinic in Mumbai (formerly Bombay), India Int J STD AIDS 2000 11 1 45 8 10.1258/0956462001914896 10667900

[B13] Jana S Bandyopadhyay N Mukherjee N STD/HIV intervention in sex workers in West Bengal India AIDS 1998 12 SB S101 S8 9679635

[B14] Urmil AC Dutta PK Basappa K A study of morbidity pattern among prostitutes attending a municipal clinic in Pune Journal of Indian Medical Association 1989 87 29 31 2789264

[B15] IHAA Frontiers Prevention Project: Participatory Site Assessments in Cambodia, Ecuador and Andhra Pradesh State in India Brighton, UK 2003

[B16] Hausman JA Specification Tests in Econometrics Econometrica 1978 46 6 1251 71 10.2307/1913827

[B17] Weiss H Buvé A Robinson N Van Dyck E Kahindo M Anagonou S The epidemiology of HSV-2 infection and its association with HIV infection in four urban African populations AIDS 2001 15 S4 S97 S108 10.1097/00002030-200108004-00011 11686471

[B18] Freeman E Orroth K White R Glynn J Bakker R Boily M Proportion of new HIV infections attributable to herpes simplex 2 increases over time: simulations of the changing role of sexually transmitted infections in sub-Saharan African HIV epidemics Sex Transm Infect 2007 83 S1 i17 i24 10.1136/sti.2006.023549 17405782

[B19] Clark J Konda K Munayco C Pún M Lescano A Leon S Prevalence of HIV, herpes simplex virus-2, and syphilis in male sex partners of pregnant women in Peru BMC Public Health 2008 8 65 10.1186/1471-2458-8-65 18284696 PMC2265685

[B20] Freedman E Mindel A Epidemiology of herpes and HIV co-infection Journal of HIV Therapy 2004 9 1 4 8 15071423

[B21] Ramesh BM Beattie TSH Shajy I Washington R Jagannathan L Reza-Paul S Changes in risk behaviours and prevalence of sexually transmitted infections following HIV preventive interventions among female sex workers in five districts in Karnataka state, south India Sexually Transmitted Infections 2010 86 Suppl 1 i17 i24 10.1136/sti.2009.038513 20167725 PMC3252604

[B22] Kumta S Lurie M Weitzen S Jerajani H Gogate A Row-Kavi A Bisexuality, Sexual Risk Taking, and HIV Prevalence Among Men Who Have Sex With Men Accessing Voluntary Counseling and Testing Services in Mumbai, India J Acquir Immune Defic Syndr 2009 10.1097/QAI.0b013e3181c354d8 PMC2844633 19934765

[B23] Brahmam GN Kodavalla V Rajkumar H Rachakulla HK Kallam S Myakala SP Sexual practices, HIV and sexually transmitted infections among self-identified men who have sex with men in four high HIV prevalence states of India Aids 2008 22 Suppl 5 S45 57 10.1097/01.aids.0000343763.54831.15 19098479

[B24] Setia MS Brassard P Jerajani HR Bharat S Gogate A Kumta S Men who have sex with men in India: a systematic review of the literature J LGBT Health Res 2008 4 2-3 51 70 10.1080/15574090902913727 19856739

